# RNF34 overexpression exacerbates neurological deficits and brain injury in a mouse model of intracerebral hemorrhage by potentiating mitochondrial dysfunction-mediated oxidative stress

**DOI:** 10.1038/s41598-019-52494-x

**Published:** 2019-11-08

**Authors:** Xin Qu, Ning Wang, Wenjin Chen, Meng Qi, Yueqiao Xue, Weitao Cheng

**Affiliations:** Department of Neurosurgery, Xuan Wu Hospital, Capital Medical University, Beijing, 100053 China

**Keywords:** Ubiquitylation, Molecular neuroscience

## Abstract

Intracerebral hemorrhage (ICH) is a common neurological condition associated with high disability and mortality. Alterations in protein ubiquitination have emerged as a key mechanism in the pathogenesis of neurological diseases. Here, we investigated the effects of the E3 ubiquitin ligase ring finger protein 34 (RNF34) on neurological deficits and brain injury in ICH mice. An ICH model was established via intracerebral injection of autologous blood into wild-type and RNF34 transgenic mice. Brain injury, neurological function, neuronal activity, and oxidative stress levels were measured, respectively. The underlying mechanisms were explored by molecular and cellular approaches. Our results showed that RNF34 overexpression in mice significantly aggravated the ICH-induced memory impairment, brain edema, infarction, hematoma volume, and loss of neuronal activity. RNF34 and oxidative stress levels gradually increased from 6 to 48 h after the ICH challenge and were positively correlated. The ICH-induced increase in intracellular ROS, superoxide anion, and mROS generation and the decrease in adenosine triphosphate production were exacerbated in RNF34 transgenic mice, but NADPH oxidase activity was unaffected. Moreover, RNF34 upregulation potentiated the ICH-induced decrease in PGC-1α, UCP2, and MnSOD expressions. RNF34 interacted with PGC-1α and targeted it for ubiquitin-dependent degradation. This study reveals that RNF34 exacerbates neurological deficits and brain injury by facilitating PGC-1α protein degradation and promoting mitochondrial dysfunction-mediated oxidative stress.

## Introduction

Stroke is the most widespread cardiovascular disease and a leading cause of disability^1^. Nontraumatic intracerebral hemorrhage (ICH) is a subtype of stroke caused by bleeding within the brain parenchyma^1,2^. Although ICH only accounts for 20–30% of stroke cases in Asia, its mortality rate is still higher than that of ischemic stroke^3^. Indeed, ICH patients show 1-year mortality rates up to 60%^3^. Thus, there is an urgent need for an understanding of ICH pathophysiology and effective treatment options after ICH injury.

Previous studies suggested that hematoma can induce glutamate release and subsequently cause oxidative stress and mitochondrial dysfunction, which play an important role in brain injury after ICH^4–7^. Oxidative stress resulting from inadequate scavenging or excessive generation of reactive oxygen species (ROS) triggers protein damage, neuronal apoptosis, and neuroinflammation, which lead to neurological deterioration in ICH patients^6^. Moreover, increased ROS attenuates GABAergic inhibitory inputs to the rostral ventrolateral medulla, contributing to sympathoexcitation and hypertension^8^. ROS is derived from several sources, including NADPH oxidase, xanthine oxidase and mitochondria^9,10^. In addition, the mitochondrial dysfunction contributes to an adenosine triphosphate (ATP) deficiency and excessive ROS generation, which further damage mitochondria and aggravate brain injury^5,7^. Hence, management of oxidative stress and mitochondrial dysfunction may be an effective therapeutic strategy for brain injury after ICH.

Ubiquitination plays an important role in regulating ROS homeostasis, participating in the control of ROS production and clearance^11^. RNF34 is an E3 ubiquitin ligase that regulates protein substrates by post-translational modification^12^. It is associated with regulating postsynaptic γ2-GABA_A_R clustering and GABAergic synaptic innervation in hippocampal neurons^13^, indicating the importance of RNF34 in regulating neurological function. However, apart from this study, the information related to RNF34 in nervous system is very limited. Further, RNF34 has been shown to be an E3 ubiquitin ligase targeting for peroxisome proliferator-activated receptor gamma coactivator 1-α (PGC-1α), which is critical for oxidative stress, mitochondrial biogenesis and cell metabolism^12,14^. However, the association between RNF34 and oxidative stress, and the possible role of RNF34 in regulating the development of ICH, remain unclear.

In this study, we observed marked phenotypes in RNF34 transgenic mice after ICH surgery, as evidenced by aggravated neurological deficits. We hypothesize that RNF34 potentiates neurological deficits and brain injury induced by ICH through oxidative stress and mitochondrial dysfunction. Our findings suggest that RNF34 may be a novel molecular target for the treatment of ICH.

## Results

### RNF34 overexpression exacerbates ICH-induced neurological deficits

**Figure 1:**
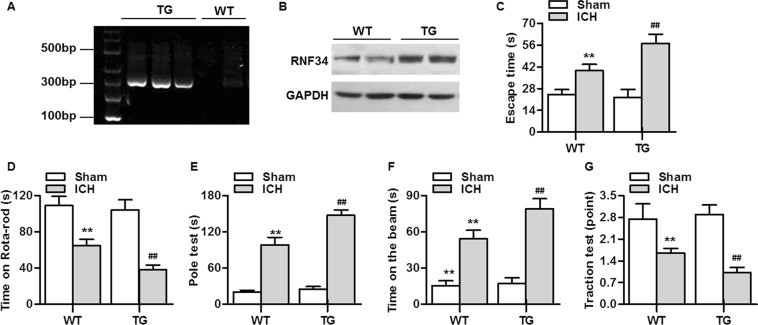
RNF34 upregulation in mice exacerbates intracerebral hemorrhage (ICH)-induced neurological deficits. (**A**) The genotypes of wild-type (WT) and RNF34 transgenic (TG) mice were determined by PCR analysis of DNA isolated from tail biopsies. (**B**) Western blotting of RNF34 expression in brain tissues of WT and TG mice. (**C**–**G**) ICH model was established as described in Method section. Summarized data of neurological deficits as assessed by Morris water maze test (**C**), rotarod test (**D**), pole test (**E**), beam-crossing task (**F**), and traction test (**G**). **P < 0.01 vs. WT sham; ^##^*P* < 0.01 vs. WT ICH, n = 20/group.

As displayed in Fig. 1A, the expression of RNF34 DNA was determined with PCR genotyping. We also determined the expression of RNF34 protein in brain tissues, finding that RNF34 expression was increased in RNF34 transgenic mice compared with wild-type mice, as expected (Fig. 1B). To investigate the role of RNF34 in ICH, we initially compared neurobehavioral outcomes between wild-type and transgenic mice. The Morris water maze test revealed that transgenic mice took a similar time to find the escape platform as wild-type mice. ICH challenge significantly extended the time for both wild-type and transgenic mice. However, the memory impairment was greater in transgenic mice than in wild-type mice, as evidenced by the longer time required to reach the platform (Fig. 1C). We next analyzed the effect of RNF34 on motor function using four kinds of behavioral tests, including the rotarod test, pole test, beam-crossing task, and traction test. The results of the rotarod test showed that ICH significantly impaired the performance of the mice, which was more pronounced in transgenic mice (Fig. 1D). The pole test revealed that the total locomotor activity was markedly increased after ICH surgery, with the locomotor activity increase further potentiated in transgenic mice (Fig. 1E). Moreover, ICH increased the time required for the mouse to cross the beam, which was also prolonged after RNF34 upregulation (Fig. 1F). Finally, the ICH-induced impairment of limb movement was further aggravated in transgenic mice (Fig. 1G).

### RNF34 upregulation aggravates the ICH-induced brain injury

**Figure 2:**
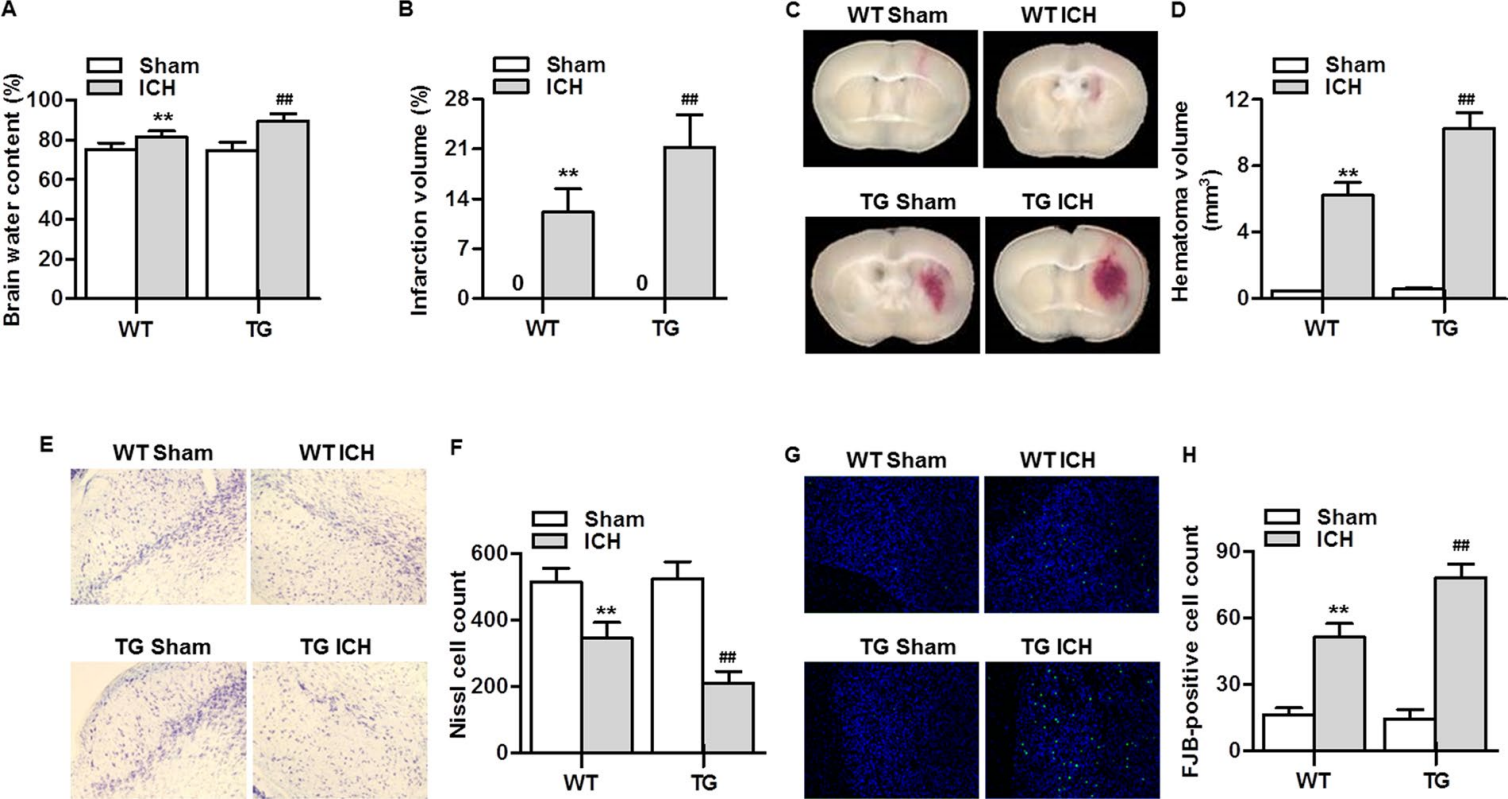
RNF34 overexpression aggravates ICH-induced brain injury. (**A**) Brain water content was measured on day 2 post-ICH. n = 5/group. (**B**) The brain infarction was determined by TTC staining and quantitative analysis of the total infarct volume. n = 5/group. (**C**) Hematomas on day 2 after ICH were measured for size. Representative images from fresh brain coronal sections. (**D**) Quantification of hematoma volume. n = 5/group. (**E**) Representative images of Nissl staining in the brain tissues. (**F**) Quantitative analysis of nissl cell. n = 6/group. (**G**) The brain sections were stained with Fluoro-Jade B (FJB) and the representative images were shown. (**H**) Quantitative analysis of FJB-positive cells. n = 6/group. **P < 0.01 vs. WT sham; ^##^*P* < 0 0.01 vs. WT ICH.

To explore whether the increased dyskinesia in RNF34 transgenic mice was the result of more severe brain injury, we examined brain edema, infarction, and neuronal activity. As shown in Fig. 2A, ICH evoked a marked elevation in brain water content in both wild-type and transgenic mice. Compared with wild-type mice, transgenic mice showed significantly increased brain edema. TTC staining revealed that there was no detectable infarct in sham wild-type and transgenic mice. Two days after ICH surgery, excessive infarction was detected, but the extent of the increase was more noticeable in transgenic mice (Fig. 2B). Moreover, ICH consistently produced significant intrastriatal hematoma in mice (Fig. 2C). Following analysis of the quantification, the transgenic mice had a greater lesion volume than the wild-type mice 48 h post ICH (Fig. 2D). ICH also led to a decrease in Nissl substance dyed purple compared with sham mice. The neuronal density loss was markedly potentiated in transgenic mice (Fig. 2E,F). We also observed a marked rise in the number of FJB-positive neurons after ICH surgery, indicating an increase in degenerating neurons. The increase in FJB-positive neurons was further enhanced in transgenic mice (Fig. 2G,H). These results were in agreement with those of Nissl staining.

### RNF34 expression parallels the ICH-induced increase in oxidative stress

**Figure 3:**
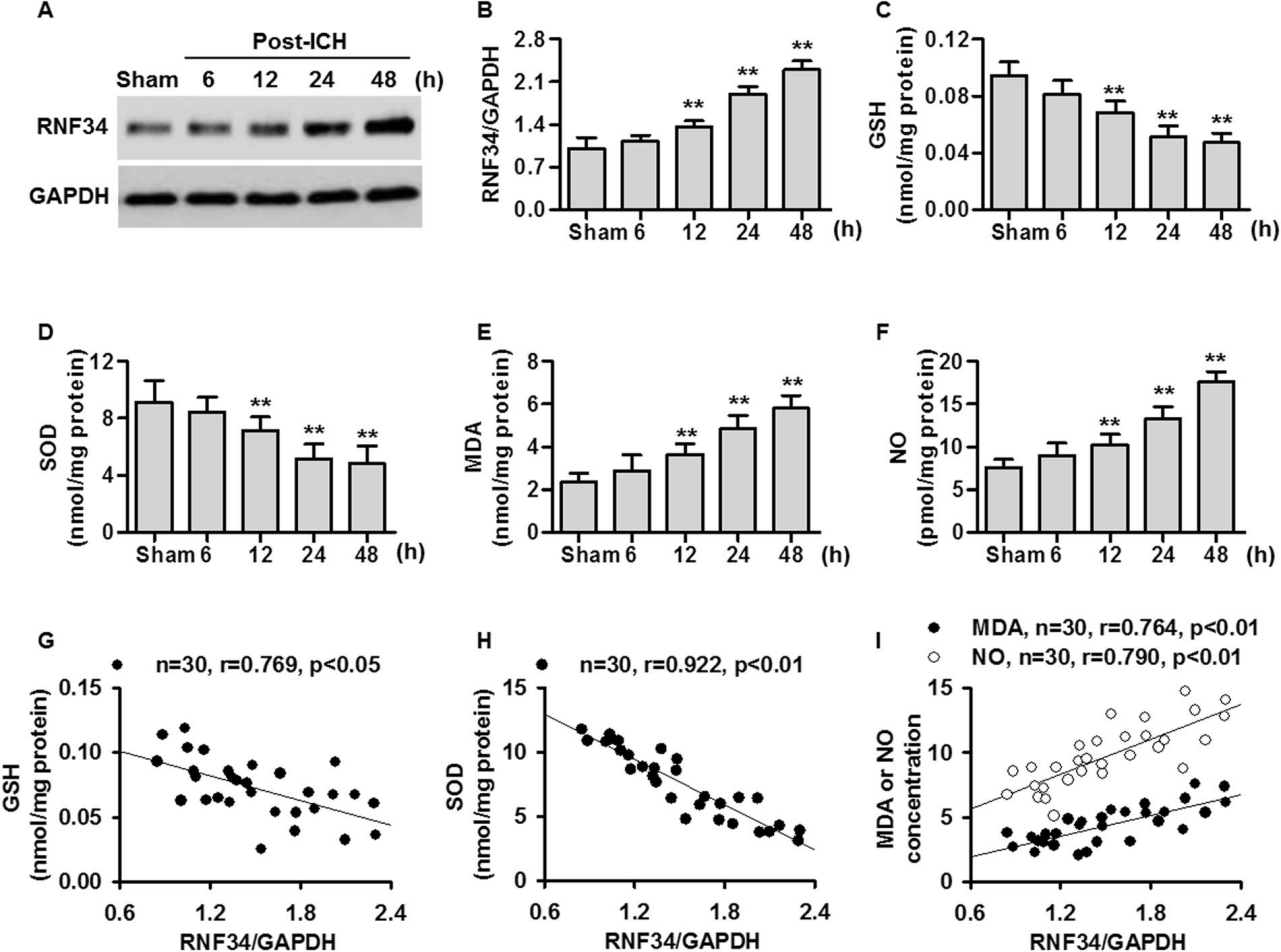
ICH increases RNF34 expression and induces oxidative stress in mouse brain. (**A**) RNF34 expression in mouse brain injury area was determined by western blotting on 6, 12, 24, and 48 h after ICH surgery. (**B**) Quantitative analysis of RNF34 expression after normalization against GAPDH. n = 6/group. (**C**–**F**) The levels of oxidative stress indicators glutathione (GSH) (**C**), superoxide dismutase (SOD) (**D**), malondialdehyde (MDA) (**E**), and nitric oxide (NO) (**F**) in brain were examined. n = 6/group. (**G**–**I**) RNF34 protein expression was compared with brain levels of GSH (G), SOD (H), MDA (**I**), and NO (**I**). n = 30 mice from 5 groups. **P < 0.01 vs. sham.

Western blotting results showed that RNF34 expression was gradually increased after ICH surgery from 6 to 48 h compared with sham mice (Fig. 3A,B). Additionally, the levels of GSH and SOD were gradually decreased 6, 12, 24, and 48 h after ICH surgery, whereas the MDA and NO levels were increased in a time-dependent manner (Fig. 3C–F). Interestingly, the RNF34 expression increase was negatively correlated with GSH and SOD levels but positively correlated with MDA and NO levels (Fig. 3G–I). These findings demonstrate that RNF34 expression is significantly correlated with the level of oxidative stress, suggesting that RNF34 may be involved in ICH-induced oxidative stress.

### RNF34 overexpression potentiates the ICH-induced oxidative stress and mitochondrial dysfunction

**Figure 4:**
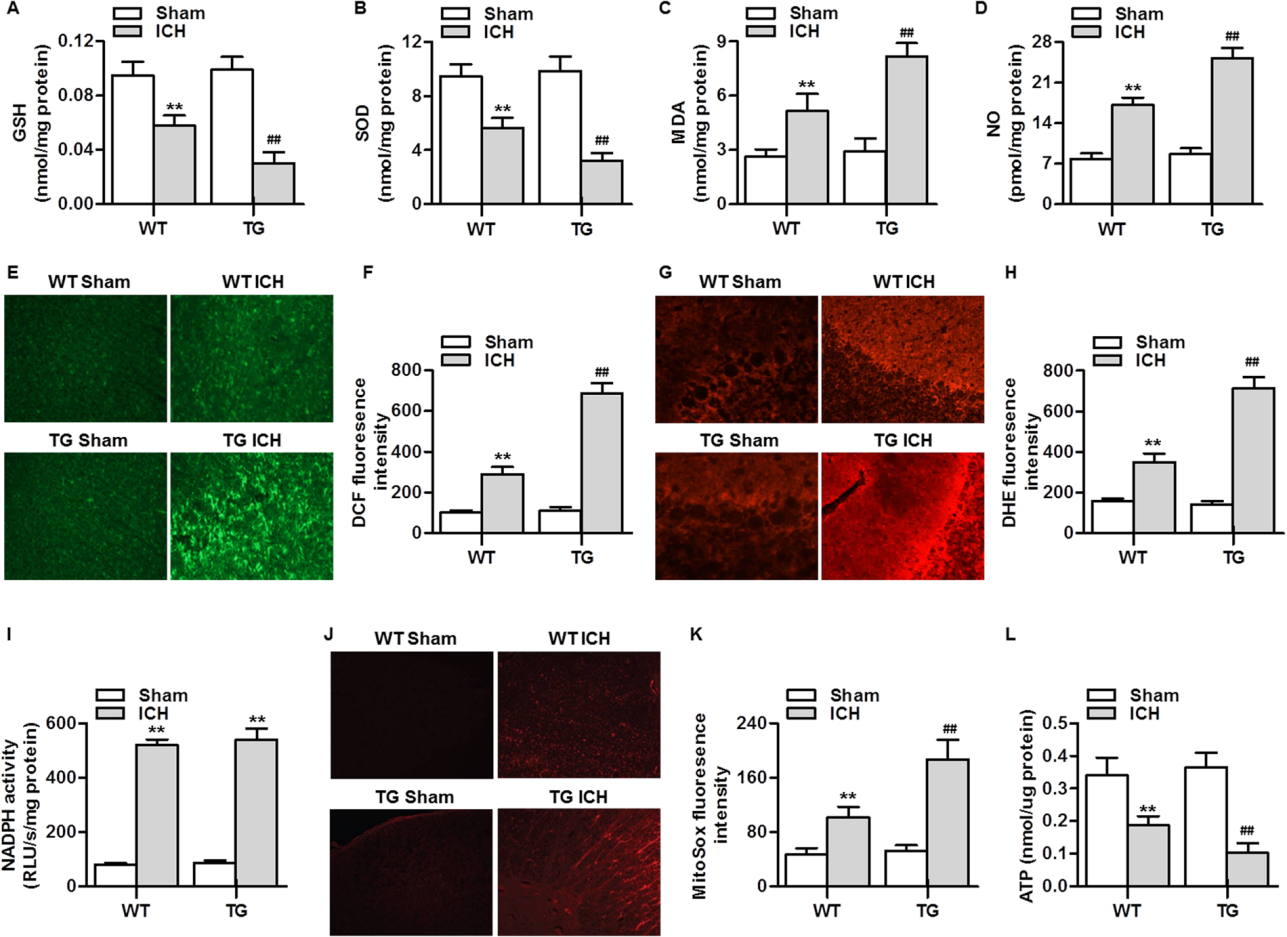
RNF34 overexpression potentiates the ICH-induced mitochondrial ROS generation in mouse brain. (**A**–**D**) The levels of GSH (**A**), SOD (**B**), MDA (**C**), and NO (**D**) in brain tissues from WT and RNF34 transgenic mice with sham or ICH surgery was determined. n = 6/group. (**E**) ROS generation in brain sections was determined by H_2_DCF-DA staining. (**F**) Quantitative analysis of DCF fluorescence intensity. (**G**) Superoxide anion was examined by dihydroethidium (DHE) staining. (**H**) Quantitative evaluation of DHE fluorescence intensity was performed. n = 4/group. (**I**) NADPH oxidase activity was measured. n = 6/group. (**J**) Mitochondrial ROS generation was determined using MitoSOX Red staining. (**K**) Quantitative evaluation of MitoSOX fluorescence intensity. n = 5/group. (**L**) ATP activity was measured. n = 6/group. **P < 0.01 vs. WT sham; ^##^*P* < 0.01 vs. WT ICH.

To determine the effect of RNF34 on ICH-induced oxidative stress, we examined the levels of GSH, SOD, MDA, and NO in the brains of wild-type and transgenic mice. No significant difference was observed in the level of oxidative stress between sham wild-type and transgenic mice. After ICH surgery, the levels of GSH and SOD were markedly lower in transgenic mice than in wild-type mice. Furthermore, the increase in MDA and NO levels was more pronounced in transgenic mice than in wild-type mice (Fig. 4A–D). Intracellular ROS generation assessed with H_2_DCF-DA was also increased after ICH surgery; this increase was markedly potentiated in transgenic mice (Fig. 4E,F). As expected, RNF34 upregulation furthered the ICH-induced increase in superoxide anion generation, as detected with DHE staining (Fig. 4G,H). To distinguish the involvement of two major sources of ROS generation—NADPH oxidase and mitochondria-derived ROS—in RNF34-potentiated ROS generation, we tested NADPH activity and mROS generation, respectively. Although ICH resulted in a marked increase in NADPH activity, no significant difference was observed between wild-type and transgenic mice (Fig. 4I). However, RNF34 overexpression significantly augmented the ICH-induced increase in mROS generation (Fig. 4J,K), indicating the occurrence of mitochondrial dysfunction. Accordingly, the ATP level was examined. ICH led to a significant decrease in the ATP level, and this decrease was more pronounced in transgenic mice than in wild-type mice (Fig. 4L). Taken together, these findings suggest that RNF34 plays an important role in regulating mitochondrial oxidative stress and function.

### mROS inhibition attenuates the effects of RNF34 overexpression on ICH-induced neurological deficits and oxidative stress

**Figure 5:**
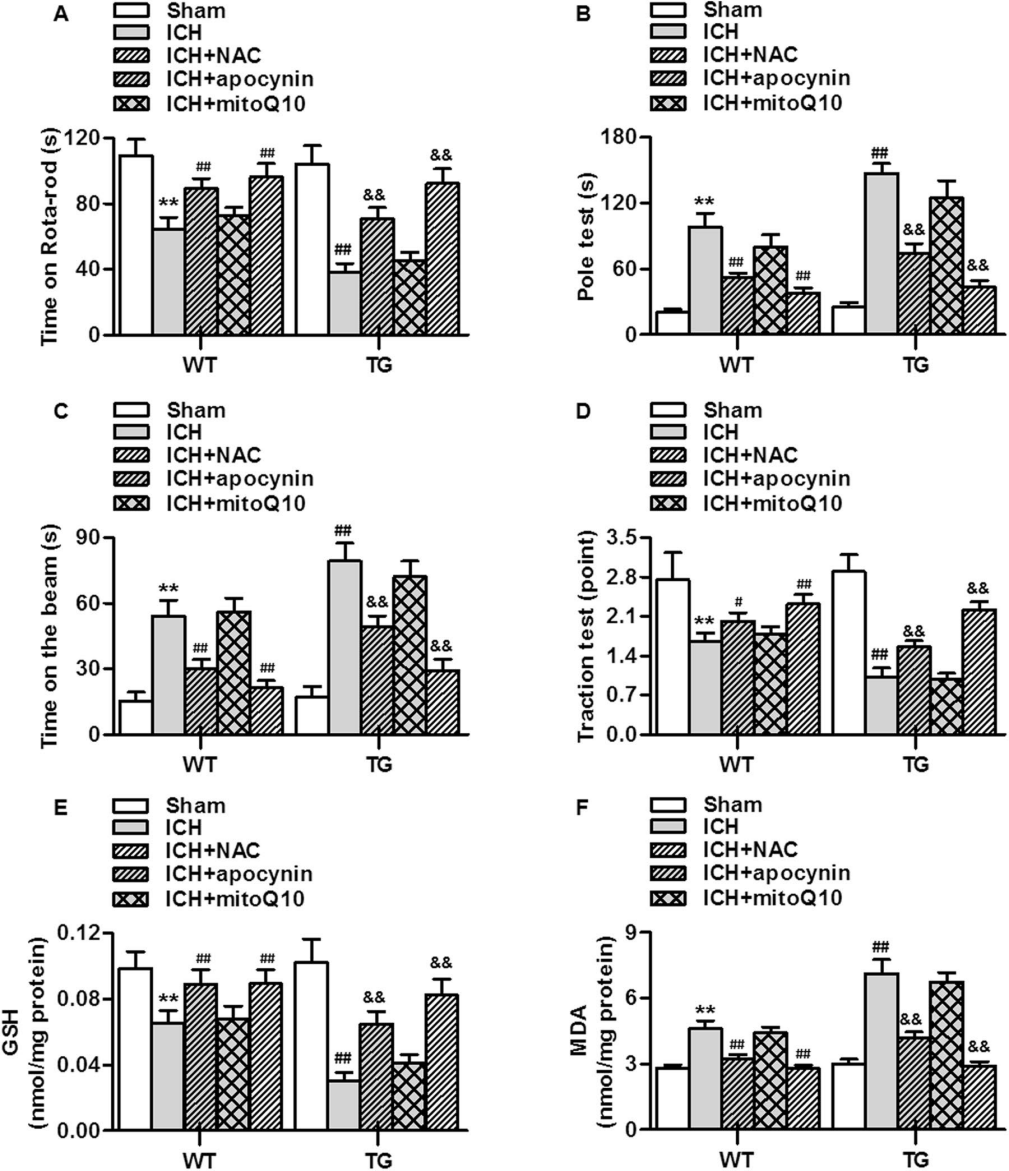
Inhibition of mitochondrial ROS generation improves the ICH-induced neurological deficits. (**A**–**D**) N-acetylcysteine (NAC), apocynin or mitoQ10 was intraperitoneally injected at a dose of 5, 20 or 10 mg/kg immediately after ICH surgery in WT and RNF34 transgenic mice. Summarized data of neurological deficits as assessed by rotarod test (**A**), pole test (**B**), beam-crossing task (**C**), and traction test (**D**). (**E**,**F**) The oxidative stress indicators GSH (**E**) and MDA (**F**) concentrations in brain tissues were determined. n = 6 per group. **P < 0.01 vs. WT sham; ^#^*P* < 0.05, ^##^*P* < 0.01 vs. WT ICH; &&*P* < 0.01 vs TG ICH, n = 20/group.

To verify the role of mROS in the RNF34-potentiated neurological deficits in ICH mice, the mitochondria-specific antioxidant mitoQ10 was used. Administration of mitoQ10 significantly reversed the poorer performance of transgenic mice in the rotarod test, pole test, beam-crossing task, and traction test. Antioxidant NAC treatment also attenuated the effect of RNF34 overexpression, similar to mitoQ10 treatment. In sharp contrast, inhibition of NADPH oxidase activity using the specific inhibitor apocynin had no effect on the ICH-induced dyskinesia in both wild-type and transgenic mice (Fig. 5A–D). Moreover, the enhanced effects of RNF34 on oxidative stress were markedly abolished by mROS inhibition but not NADPH inhibitor (Fig. 5E,F). These data indicate that the enhanced mROS generation at least contributes to the potentiated effects of RNF34 on neurological deficits.

### RNF34 overexpression promotes PGC-1α protein degradation

**Figure 6:**
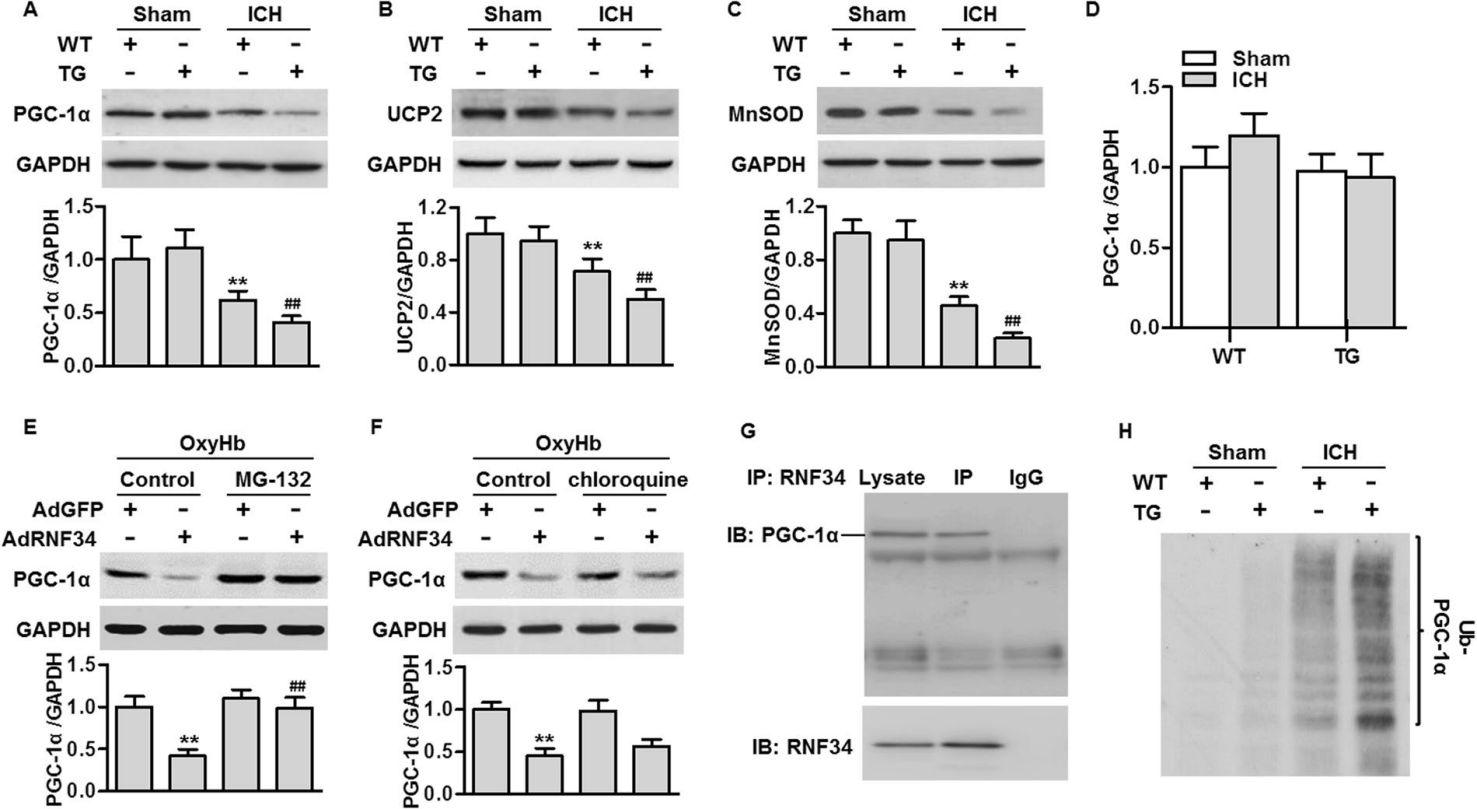
RNF34 interacts with PGC-1α and promotes ICH-induced PGC-1α ubiquitination in brain tissues. (**A**–**C**) The expression of PGC-1α (**A**), UCP2 (**B**) and MnSOD (**C**) were detected with western blotting. **P < 0.01 vs. WT sham; ^##^*P* < 0.01 vs. WT ICH, n = 6/group. (**D**) Real-time PCR analysis of PGC-1α mRNA expression. (E and F) Western blotting of PGC-1α expression in neurons pretreated with MG132 (10 μg/mL) (**E**) or chloroquine (10 μg/mL) (**F**) for 30 min in prior to RNF34 or GFP adenovirus treatment (50 MOI) in the presence of oxyhemoglobin (OxyHb, 10 μmol/L) for 48 h. **P < 0.01 vs. OxyHb + AdGFP; ^##^P < 0.01 vs. OxyHb + AdRNF34, n = 6. (**G**) Brain lysates were immunoprecipitated (IP) with RNF34 antibody and immunoprecipitated proteins were immunoblotted with PGC-1α antibody. (**H**) Brain lysates from each group were immunoprecipitated with PGC-1α antibody and blotted with ubiquitin antibody. n = 5/group.

PGC-1α plays a critical role in regulating mitochondrial biogenesis and oxidative stress and is a suggested target of RNF34^14,15^. We next investigated the effects of RNF34 on PGC-1α expression. Western blotting results showed that ICH significantly decreased RNF34 expression in the brain, concomitantly with reduced expressions of its downstream genes UCP2 and MnSOD. The decrease in these above gene expressions was even greater in transgenic mice (Fig. 6A–C). To clarify how RNF34 decreases PGC-1α expression under ICH conditions, we first determined the PGC-1α mRNA level. Notably, the PGC-1α mRNA level was unchanged among the groups (Fig. 6D). These data exclude the possibility that RNF34 inhibits PGC-1α expression via transcriptional regulation. Considering that RNF34 is an E3 ubiquitin ligase that has been suggested to interact with PGC-1α^12^, we next determined whether RNF34 is involved in PGC-1α protein degradation. The inhibitory effect of RNF34 on PGC-1α expression in neurons was markedly abolished by the proteasome inhibitor MG132, but not the lysosome blocker chloroquine (Fig. 6E,F), suggesting that RNF34 inhibits PGC-1α expression through the proteasome degradation pathway. Consistent with previous results^12^, immunoprecipitation clearly showed that RNF34 interacted with PGC-1α (Fig. 6E). Importantly, ICH increased the level of PGC-1α ubiquitination, and the increase in PGC-1α ubiquitination was potentiated in transgenic mice (Fig. 6F). The above results suggest that RNF34 promotes the ICH-induced decrease in PGC-1α expression by facilitating ubiquitin-mediated degradation.

## Discussion

This unveils a link between RNF34 and mitochondrial dysfunction-mediated oxidative stress in the pathogenesis of ICH. We provide the first evidence that RNF34 exacerbates oxidative stress, brain injury, and dyskinesia by reducing PGC-1α expression and potentiating mitochondrial dysfunction in the ICH mouse model.

Emerging evidences indicates that pathological alteration of protein ubiquitination plays a crucial role in the development of neurological diseases^16,17^. For example, tumor necrosis factor receptor-associated factor 6 (TRAF6) ubiquitinates Rac1 and worsens ischemic stroke^18^. Nedd4–2 directly targets glutamate transporter and inhibits glutamate uptake in a Parkinson’s disease model^19^. Moreover, RNF167 negatively regulates synaptic AMPAR expression and currents, which are crucial regulators of synaptic transmission and plasticity^17^. Although RNF34 has been shown to decrease synaptic innervation via ubiquitination of the γ2-GABA_A_R subunit^13^, the role of RNF34 in neurological diseases is poorly understood. Here, we investigated the alteration in RNF34 in brain tissues. The results showed that RNF34 expression was gradually decreased after ICH surgery, indicating that RNF34 may play a role in the pathogenesis of ICH. More importantly, we found that RNF34 transgenic mice displayed more severe memory impairment after ICH surgery than wild-type mice, although RNF34 upregulation had no clear phenotype under normal physiological conditions. The mice showed poor performances in behavioral tests. Brain edema and infarction are the main pathological changes in ICH and the leading cause of death^20,21^. The results showed that RNF34 overexpression significantly increased brain edema and infarction, suggesting that RNF34 prevents recovery from the blood–brain barrier breakdown. Additionally, Nissl and FJB staining revealed that the loss of neuronal activity was markedly augmented in RNF34 transgenic mice. These findings demonstrate that RNF34 may appear to play a neuroprotective role after a challenge such as ICH.

Oxidative stress is widely implicated in the pathogenesis of neurological diseases^4,7,15^. Notably, the extent of the RNF34 expression increase was correlated with oxidative stress in the brain, indicating the involvement of RNF34 in oxidative stress after ICH surgery. Indeed, after ICH challenge, the increase in oxidative stress was significantly augmented in transgenic mice. Because NADPH oxidase and the mitochondrial electron transport chain are the major sources of intracellular ROS^9,10^, we next determined the effects of RNF34 on NADPH oxidase activity and mROS generation. We showed that the ICH-induced increase in NADPH oxidase activity was comparable in wild-type mice and transgenic mice. However, the increase in mROS generation was promoted by RNF34 overexpression. These findings suggest that mitochondria-derived ROS generation is responsible for the increase in oxidative stress after RNF34 upregulation. Moreover, the subsequent results showed that mROS inhibition but not NADPH oxidase inhibition ameliorated the neurological deficits in transgenic mice, further supporting the belief that mitochondria-derived ROS generation is critical for the ability of RNF34 to promote neurological dysfunction.

Dysregulation of mitochondria biogenesis is considered a major contributor to mitochondrial dysfunction, which leads to sustained oxidative stress^4,10^. The critical role of mitochondrial biogenesis in controlling mitochondrial function is supported by the evidence that calorie restriction can increase mitochondrial biogenesis and lifespan, with lower oxidative stress despite high ATP production^22^. Our results showing that RNF34 overexpression potentiated oxidative stress and concomitantly decreased ATP level are in full agreement with this theory. PGC-1α has been well documented to specifically regulate mitochondrial biogenesis and cell metabolism^12,15^. PGC-1α knockout can induce mitochondrial oxidative stress and vascular inflammation^23^. Our results showed that RNF34 overexpression potentiated the ICH-induced the inactivation of PGC-1α and decreased the levels of the downstream oxidative stress-protective molecules UCP2 and MnSOD, promoting mitochondrial dysfunction.

Surprisingly, we found that RNF34 had no effects on PGC-1α mRNA expression, suggesting that transcriptional regulation is not involved. Recently, posttranslational modification emerged as another important mechanism regulating PGC-1α expression and function. Considering that RNF34 is an E3 ubiquitin ligase^12^, we explored whether RNF34 regulates PGC-1α expression via ubiquitin-dependent degradation. Several E3 ligases have been implicated in the regulation of PGC-1α expression, such as SCF^Fbw7^ and Mule^24,25^. In the ubiquitination system (E1 through to E3), E3 ligases are of particular interest because they determine substrate specificity and the transfer of ubiquitin to target proteins^26,27^. Notably, previous work showed that RNF34 binds to PGC-1α and mediates its degradation in brown fat cells^12^. Consistent with these findings, we found that RNF34 interacts with PGC-1α in brain tissues. RNF34 overexpression markedly enhanced the ICH-induced the ubiquitination of PGC-1α. Thus, RNF34 decreases PGC-1α expression probably via ubiquitin-mediated degradation.

In summary, our results demonstrate that RNF34 facilitates the ubiquitin-mediated degradation of PGC-1α, which in turn aggravates brain injury and neurological deficits by promoting mitochondrial dysfunction and subsequent oxidative stress. These findings suggest that genetic targeting of RNF34 may be a promising strategy for the treatment of ICH.

## Materials and Methods

### Reagent and materials

N-acetylcysteine (NAC), apocynin, mitoQ10, triphenyltetrazolium chloride (TTC), Fluoro-Jade B (FJB), 2′,7′-dichlorofluorescin diacetate (H_2_DCF-DA), dihydroethidium (DHE), MitoSOX Red, NADPH, lucigenin, MG-132, chloroquine and oxyhemoglobin (OxyHb) were obtained from Sigma (St. Louis., MO). Nissl staining solution, RIPA lysis buffer and horseradish peroxidase (HRP)-conjugated secondary antibodies were purchased from Beyotime (Jiangsu, China).

### Generation of transgenic RNF34 mice

Male wild-type C57/BL6 mice were purchased from the Jackson Laboratory (Bar Harbor, ME). Transgenic mice were generated using a pRP.ExBi-CMV-IRES expression vector. The RNF34 gene was used as template DNA and cloned into the CMV promoter, which was subsequently microinjected into C57/BL6 background mice. Founder mice were then backcrossed to wild-type mice to generate transgenic offspring. The genotypes were confirmed by PCR analysis of DNA isolated from tail biopsies using the following primers—sense, 5′-AGGGGATGAC CTGGACTCAA-3′ and antisense, 5′-AGGTCAGACA GGGAAGCTCT-3′—, which resulted in a 320-bp fragment of RNF34 cDNA. All animal experiments were performed in accordance with China Animal Welfare Legislation and approved by the Institutional Animal Care and Use Committee of Capital Medical University.

### ICH model

An autologous blood infusion model was used to stimulate ICH *in vivo* as previously described^2,28,29^. Briefly, mice were anesthetized with 3% isoflurane in 67% N_2_O/30% O_2_ and fixed on a mouse stereotaxic frame (Precision Systems and Instrumentation, Fairfax Station, VA). A 0.5-mm burr hole was created using a drill in the skull (2.0 mm right lateral to the midline and 0.2 mm anterior to bregma). Twenty-five microliters of autologous blood collected from the central tail artery was infused stereotactically through the hole into the right striatum via a 30-gauge microsyringe in two stages. First, 5 μL of autologous blood was delivered at a rate of 2 μL/min using a microinfusion pump (TJ-1A, LongerPump, Shanghai, China). Five minutes later, the remaining 20 μL was infused at the same rate. The microsyringe was carefully removed, the hole was sealed, and the skin incision was closed. The mouse body temperature was maintained by a 37 °C heat lamp throughout the procedure from the start of the surgery until the animals recovered from anesthesia. Sham mice received the same procedure without autologous blood infusion. Mice that died during the surgery were excluded.

### Experimental procedure

Mice were randomly divided into the following groups: wild-type sham (n = 25), wild-type ICH (n = 80), wild-type ICH + NAC (n = 20), wild-type ICH + apocynin (n = 20), wild-type ICH + mitoQ10 (n = 20), transgene sham (n = 25), transgene ICH (n = 25), transgene ICH + NAC (n = 20), transgene ICH + apocynin (n = 20) and transgene ICH + mitoQ10 (n = 20). NAC, apocynin or mitoQ10 was intraperitoneally injected at a dose of 5, 20, or 10 mg/kg, respectively, immediately after ICH surgery and then continuously dosed once a day for 2 days. To examine the changes in RNF34 expression after ICH challenge, the wild-type ICH group was further divided into four subgroups according to time point (6 h [n = 20], 12 h [n = 20], 24 h [n = 20], and 48 h [n = 25]). No mice died during the experimental period.

### Primary rat cortical neuron cultures and adenoviral infection

Primary neurons were extracted from newborn Wistar rats (Animal Center of Capital Medical University) s described previously^30^. Briefly, rats were decapitated, their cortices were dissected in ice-cold Hanks Buffered Salt Solution, and the cells were dissociated by trypsinization. Two hours later, adherent cells were cultured in Dulbecco’s Modified Eagles Medium at 37 °C with 5% CO_2_. The primary cortical neurons were exposed to OxyHb to mimic ICH *in vitro*. Rat RNF34 adenovirus (AdRNF34) and negative control (AdGFP) were constructed by Cyagen (Santa Clara, CA). The adenovirus was diluted in medium without serum, which was then gently added to the cells. After 3 h, the cells were transferred into fresh culture medium and incubated for 48 h before analysis.

### Western blotting and immunoprecipitation

The perihematoma tissues were gathered and protein was extracted by RIPA lysis buffer. Protein concentration in the samples was determined by a bicinchoninic acid kit (Bio-Rad, Hercules, CA). Equal protein (100 μg) was loaded and separated by 8% SDS-PAGE gels, and transferred onto polyvinylidene fluoride membranes (Millipore, Billerica, MA) at 200 mA for 90 min. After blocking by 5% non-fat milk, the membranes were incubated by the following primary antibodies at 4 °C overnight: RNF34 (1:500) (OriGene Technologies, Beijing, China), PGC-1α (1:800) (Abcam, Cambridge, MA), uncoupling protein 2 (UCP2), manganese-dependent superoxide dismutase (MnSOD) and GAPDH (1:1000) (Santa Cruz Biotechnology, Santa Cruz, CA), followed by incubation with HRP-conjugated secondary antibodies for 1 h at room temperature. Detection was performed using enhanced chemiluminescence (Amersham Pharmacia, Piscataway, NJ). GAPDH was used as an internal loading control. For immunoprecipitation assay, protein lysates were pre-cleared with A/G agarose beads (Santa Cruz Biotechnology) for 2 h and then co-incubated with A/G agarose beads and RNF34 antibody at 4 °C overnight. The immunoprecipitated protein was determined by western blotting.

### Morris water maze test

Twenty mice from each group were used for neurobehavioral testing. All of the following behavioral tests were carried out by two trained experimenters who were blinded to animal grouping. The Morris water maze test was performed as previously described^31,32^. The water maze apparatus (Guangzhou Feidi Biology Technology Co., Ltd., Guangzhou, China) consisted of a black platform, circular pool, and recording system. The experimental equipment was divided into four quadrants and filled with nontoxic white pigments. A black platform (10 cm) was placed 2 cm under the water in the center of the fourth quadrant. The test was performed in a dark room. The mice were randomly placed in varying quadrants and allowed 90 s to find the platform. If a mouse failed to find the platform within 90 s, it was guided to the platform and allow to stay on it for 10 s. The mice were trained for 5 consecutive days before the ICH surgery. At the end of the experimental period, the mice were allowed to swim in the pool and the time spent in traversing the original platform position was recorded to reflect the degree of learning ability and spatial memory.

### Rotarod test

Motor balance and coordination abilities were evaluated using an automated Rotarod (Ugo Basile, Comerio, Italy). The mice were trained for 5 consecutive days at a constant speed of 40 rpm at the start of the experiment. The average latency to the first fall from the rod was recorded by magnetic trip plates. The maximum cutoff time was set as 180 s.

### Pole test

A pole test was performed according to a previously described method^29,33^. A ball (diameter, 2.5 cm) was glued on the top of a wooden pole (length, 50 cm; diameter, 1 cm). Each mouse was placed on top of the ball and allowed to climb down the pole. The average time of four tests was calculated.

### Beam-walking ability test

A beam approximately 100 cm long and approximately 2 cm wide was connected between two platforms suspended 20 cm above the ground. Each mouse was placed on the beam and the time required to cross the beam was measured. If the mouse did not cross the beam in 120 s, the time was recorded as 120 s.

### Traction test

A traction test was performed to determine the degree of limb impairment as previously described^29,33^. Mice were hung on a horizontal wire by their forepaws; 3 points were awarded if the mouse grasped the wire with two hind paws, 2 points were awarded if the mouse grasped the wire with one hind paw, and 1 point was awarded if the mouse did not grasp the wire with either hind paw. The average time of four tests was calculated.

### Brain water content

Brains were isolated from the skull and then divided into 3-mm coronal sections in the portion around the puncture point. The brain tissue samples from the ipsilateral basal ganglia were immediately weighed on an electronic analytical balance (BSAl24SCW; Sartorius Scientific Instruments, Beijing, China) to obtain the wet weight. Then, the tissues were placed onto pre-weighed silver paper and dried in an oven at 62 °C for 72 h to obtain the dry weight. Brain water content (%) was calculated as (wet weight − dry weight)/wet weight × 100.

### Brain infarction volume measurement

Brain sections were stained with 2% TTC at 37 °C for 30 min and then fixed in 10% formaldehyde neutral buffer solution overnight. The infract area (unstained) and normal area (red regions) were observed. The size of the infarct volume was measured using the “Count/Size” function of ImageJ software (version 1.41; National Institutes of Health, Bethesda, MD) and calculated as (infarct volume/contralateral hemisphere area) × 100%.

### Nissl staining

Nissl staining was performed to reveal the number of surviving neurons. Ten-micron-thick coronal sections from the rostral to the caudal portion of the damaged brain areas, which were spaced 200 μm apart, were incubated with Nissl staining solution at 40 °C for 10 min followed by washing with 95% ethyl alcohol and 70% ethyl alcohol in succession. Images were captured with a light microscope (BX51; Olympus, Tokyo, Japan). The number of stained cells was calculated with ImageJ software.

### FJB staining

FJB staining was used to estimate the number of degenerating neurons. After being washed with phosphate-buffered saline (PBS) twice, brain sections within 200 μm of the puncture were incubated with 0.06% potassium permanganate in PBS at room temperature for 10 min. After being washed with distilled water for 2 min, the sections were incubated in a 0.0004% FJB working solution for 20 min and then photographed under a fluorescence microscope (IX71; Olympus). The number of FJB-positive cells was calculated with ImageJ software.

### Oxidative stress level measurement

Perihematoma tissues were collected from each group and homogenized at the end of the experiment. The concentrations of glutathione (GSH), superoxide dismutase (SOD), malondialdehyde (MDA), and nitric oxide (NO) in the homogenate were determined by commercial kits according to the supplier’s instructions (Nanjing Jiancheng Bioengineering Institute, Nanjing, China). For the determination of total ROS generation, brain sections were incubated with H2DCF-DA (10 μmol/L) for 30 min at 37 °C in the dark. After being washed with PBS, images were captured with a fluorescence microscope. Superoxide anion generation was visualized with DHE (5 μmol/L) staining. After a 30-min incubation, the sections were observed under a fluorescence microscope. To test the generation of mitochondrial ROS (mROS), brain sections were incubated with MitoSOX Red (5 μmol/L) in the dark at 37 °C for 30 min and viewed with a fluorescence microscope. The fluorescence intensity was quantified with ImageJ software. NADPH oxidase activity was determined by a lucigenin-enhanced chemiluminescence assay as described previously^14^. Brain tissues were lysed in a modified buffer (in mol/L: 1.0 K_2_HPO4, 0.1 EGTA, and 0.15 phosphate and protease inhibitor cocktail). The homogenates were then centrifuged at 12000 × *g* for 5 min. Lucigenin (5 mmol/L) was added to the supernatant and incubated for 10 min at 37 °C in the dark. Basal relative light units (RLUs) of chemiluminescence were obtained with a luminometer (Promega, Madison, WI). The experimental RLU was obtained by the addition of NADPH (100 μmol/l) and recorded every 15 s for 20 min. NADPH oxidase activity was calculated as (total experimental RLU − total basal RLU)/(20 × 60 s)/total protein concentration.

### ATP assay

ATP synthesis was determined by ATP colorimetric assay kit (Biovision, Mountain View, CA). Brain homogenates were collected and the assay was performed in 96-well plates in accordance with manufacturer’s instructions. The optical density (O.D.) value was obtained at 570 nm using a micro-plate reader (Bio-Tek, Winooski, VT). The concentration of ATP was calculated according to the standard curve.

### Real-time PCR

The samples were homogenized in in TRIzol reagent (Invitrogen, Carlsbad, CA) and total RNA was isolated according to the manufacturer’s instructions. 1 μg of RNA was reverse-transcribed to cDNA according to the manufacturer’s instructions (Thermo Scientific, San Jose, CA). A SYBR QPCR Kit (Toyobo, Osaka, Japan) was used in associated with ABI 7500 real-time PCR system (Applied Biosystems, Foster City, CA) to detect PGC-1α mRNA expression. Mouse GAPDH was used as an endogenous control. The primer sequences were as follows: PGC-1α, 5′-TACGCAGGTCGAACGAAACT-3′ and 5′-GAAGGGGTCGCCCTTGTTC-3′; GAPDH, 5′-GGGCACGAAGGCTCATCATT-3′ and 5′-AGAAGGCTGGGGCTCATTTG-3′.

### Statistical analysis

All data were expressed as mean ± SEM. n value represented the number of replicates and was mentioned in figure legends. No animal were excluded from analysis. Quantitative analysis of cerebral hemorrhage volume was performed 48 h after ICH from 5 slides per mouse, allowing for an analysis that represented of the whole brain. Densitometry results of western blotting were quantified using ImageJ software. The regression analysis between RNF34 and oxidative stress levels was determined by the Pearson correlation test. Kruskal–Wallis test was used to analyze the behavioral data. The significant differences between 2 groups were analyzed by 2-tailed Student t test. One-way or 2-way ANOVA followed by Bonferroni multiple comparison test was used to compare differences when there were >2 treatment groups. The analysis was performed using Prism 7.01 software (GraphPad software, San Diego, CA). P < 0.05 was considered to be statistically significant.
